# Epstein-Barr Virus Sequence Variation—Biology and Disease

**DOI:** 10.3390/pathogens1020156

**Published:** 2012-11-07

**Authors:** Stelios Tzellos, Paul J. Farrell

**Affiliations:** Section of Virology, Imperial College Faculty of Medicine, Norfolk Place, London W2 1PG, UK; E-Mail: stelios.tzellos06@imperial.ac.uk (S.T.)

**Keywords:** Epstein-Barr virus, sequence variation, virus types

## Abstract

Some key questions in Epstein-Barr virus (EBV) biology center on whether naturally occurring sequence differences in the virus affect infection or EBV associated diseases. Understanding the pattern of EBV sequence variation is also important for possible development of EBV vaccines. At present EBV isolates worldwide can be grouped into Type 1 and Type 2, a classification based on the EBNA2 gene sequence. Type 1 EBV is the most prevalent worldwide but Type 2 is common in parts of Africa. Type 1 transforms human B cells into lymphoblastoid cell lines much more efficiently than Type 2 EBV. Molecular mechanisms that may account for this difference in cell transformation are now becoming clearer. Advances in sequencing technology will greatly increase the amount of whole EBV genome data for EBV isolated from different parts of the world. Study of regional variation of EBV strains independent of the Type 1/Type 2 classification and systematic investigation of the relationship between viral strains, infection and disease will become possible. The recent discovery that specific mutation of the EBV EBNA3B gene may be linked to development of diffuse large B cell lymphoma illustrates the importance that mutations in the virus genome may have in infection and human disease.

## 1. Introduction: Epstein-Barr virus (EBV) Variation and Disease?

EBV was discovered in cells from African Burkitt’s lymphoma but it was soon realized that infection with EBV is endemic worldwide. It is estimated that over 90% of the world’s population is infected with the virus. EBV is associated with several diseases whose incidence differs dramatically in different parts of the world [1]. Examples of this include high incidence of nasopharnygeal carcinoma (NPC) in Southern Chinese people, high incidence of Burkitt’s lymphoma (BL) in sub-Saharan Africa and high incidence of infectious mononucleosis in teenagers and young adults in western countries. Each of these exceptional geographic or demographic differences in disease incidence may be accounted for by other cofactors but there has long been interest in the possibility that genetic variation in the EBV in different parts of the world might play a role [2].

In recent years there have been many studies which show substantial geographic variation in the virus sequence in normal infected populations. These demonstrate the need to clarify what wild type EBV is, and how it varies in different parts of the world, so that disease specific variation can be identified. The problem has been illustrated very clearly by the recent discovery that inactivation of the EBNA-3B gene results in an EBV with a much higher B cell transforming activity and a propensity to cause Diffuse Large B cell Lymphoma (DLBCL) in a mouse model [3]. Detection of termination mutations of EBNA-3B in several human DLBCL cases indicates that this may be a mechanism of disease related to EBV variation. Sequencing EBNA-3B from a large number of cases and controls showed a high frequency of point sequence variation from the reference EBV but it is not yet clear whether those changes are functionally significant [3]. Hence, in general, to know whether there is a disease related variation, we need to answer the question “what is wild type EBV?”

## 2. Short History of EBV Sequencing

Because of the relatively large genome for a virus (175 kb), the presence of several tandem repeat arrays in the virus sequence and a prevailing impression that herpesvirus sequences tend not to vary very much, sequencing of EBV genomes was initially quite limited. The sequence of some small fragments of B95-8 EBV was published in 1982 [4,5] but the first complete EBV sequence (accession number V01555) of the B95-8 strain was published in 1984 [6]. At the time, it was the largest DNA sequence that had been determined and comprised 10% of the EMBL data library. 

Although it now seems somewhat atypical, the B95-8 strain was sequenced because it was from the only EBV cell line available that produced virus at a sufficient level to make it practical to clone the viral restriction fragments. B95-8 cells were originally derived [7] using EBV from 883L (a spontaneous human lymphoblastoid cell line from an infectious mononucleosis patient) to transform lymphocytes from the cotton top marmoset (Saguinus oedipus). EBV is secreted into the medium spontaneously giving useful amounts of infectious, transforming virus but B95-8 cells can also be induced into the lytic cycle with phorbol myristate acetate (PMA). To get the DNA for sequencing, B95-8 EBV was produced from PMA treated B95-8 cells and the EBV DNA was cloned as restriction fragments, Eco RI fragments in a cosmid vector pHC79 or Bam HI fragments in pBR322 [8]. Sequencing was by the Sanger method. 

It was already clear from restriction site mapping that there was a large (13.6 kb) deletion in B95-8 relative to other known EBV strains [9] and this sequence was subsequently obtained from cloned restriction fragments of EBV from the Raji Burkitt lymphoma cell line [10]. Fortunately the error rate in the original sequencing proved to be very low (about 1/50,000) so predictions made of the open reading frames [6] turned out to be an accurate guide to the genetic content and these have been the basis of much of the subsequent investigation of EBV. In the following 18 years, three single nucleotide errors discovered in the genome sequence were corrected [11] but the sequence has not been adjusted for loss of a small part of the repeat array in oriP [12,13] selected by cloning in plasmids. To facilitate studies on the whole viral genome, a “wild type” EBV sequence (EBVwt, AJ507799) was assembled from the corrected B95-8 and Raji sequence, the number of major internal repeat units in the sequence was reduced to 7.5 to be more typical and annotation was updated [11].

Finally, a more standard annotation of EBV wt including three additional small open reading frames that could now be recognized from sequence comparison was released by AJ Davison and PJ Farrell in 2010 as the RefSeq HHV4 (EBV) sequence NC_007605. This is the current standard reference sequence.

Key insights into EBV sequence variation came from publication of further complete EBV sequences from Africa and China. The EBV sequence DQ279927 from the AG876 African BL cell line [14] is a Type 2 EBV strain (see below). The Chinese GD1 isolate (AY961628) is from a lymphoblastoid cell line (LCL) made by immortalizing cord B cells with EBV from saliva of an NPC patient in Guangzhou [15]. The GD2 sequence (HQ020558) is a direct determination of EBV sequence from a Guangzhou NPC biopsy [16]. Another EBV sequence HKNPC1 from a Hong Kong NPC biopsy (JQ009376) has been published recently [17]. 

The methods used to determine these EBV sequences have advanced with technological development. The AG876 sequence was determined using Sanger sequencing of cosmid-cloned fragments, supplemented by PCR amplification of selected regions. GD1 and HKNPC1 were amplified as a set of PCR fragments, which were then sequenced. The GD2 sequence was obtained by selecting the EBV reads from Illumina sequencing of the whole NPC biopsy DNA and assembling the reads, linking the resulting contigs with Sanger sequencing of PCR fragments bridging the gaps. 

Recent advances in sequencing technology based originally on “next generation” Illumina technology are greatly increasing the amount of whole EBV genome data for EBV isolated from different parts of the world. Regional variation of EBV strains independent of the Type 1/Type 2 classification will be clarified and systematic investigation of the relationship between viral strains, infection and disease is becoming possible. The small amount of starting material required for determination of a complete EBV sequence by these methods makes it realistic to analyze large numbers of primary human EBV strains. The recent discovery [3] that specific mutation of the EBV EBNA3B gene may be linked to development of diffuse large B cell lymphoma indicates the importance that mutations in the virus genome may have in infection and human disease. 

## 3. Broad Aspects of Genome Variation—EBV Types, Selection Forces and Recombination

The first major variation to be identified in EBV was the Type 1 or Type 2 classification based on differences in EBNA2 [1,18,19]. The types were also known as Type A and B respectively. Most EBV genes differ by less than 5% in sequence in different isolates but EBNA2 clearly sorts into Type 1 or Type 2, with only 70% identity at the nucleotide level and 54% identity in the protein sequence. There is linked variation in EBNA3 genes [20] but the degree of sequence difference is less. Type 1 is the main EBV prevalent worldwide but in sub-Saharan Africa Type 2 EBV is equally abundant. Sequencing of Type 2 EBV from the AG876 cell line enabled a comparison between Type 1 and Type 2 EBV genomes [14]. This revealed that the two types are co-linear and very similar, with the exception of the known divergent alleles. The type variation has a clear phenotypic consequence in cell culture; Type 2 EBV is much less effective at establishing LCLs than Type 1 EBV [21]. There has been considerable progress in understanding the mechanism of this, described in detail separately below. 

The extent to which EBV can be meaningfully classified into types based on individual gene markers depends partly on the extent of inter-typic recombination that may occur. There is clear evidence for inter-typic recombination based on polymorphisms distributed along the genome [22,23,24]. A comparison of the B95-8, GD1 and AG876 sequences led to a proposal for a minimum theoretical number of recombination events that would be required to result in the current genome arrangements of those viruses [25]. Points of variation have been studied within Type 1 EBNA2 [26] but Type 1 and Type 2 EBNA2 sequences are sufficiently different to preclude their recombination within EBNA2. So the Type 1 and Type 2 EBNA2 characteristics will tend to survive irrespective of recombination that may occur elsewhere in the genome. Interpreting the significance of the persistence of these types will therefore depend on understanding the mechanism and phenotypic consequences of the type differences *in vivo*. The recent discovery that APOBEC3 cytidine deaminase RNA editing can modify EBV genomes in cell culture demonstrates another possible mechanism for generating virus heterogeneity *in vivo*, in addition to infection with multiple strains and virus recombination [27].

Factors that affect *in vivo* selection of viral recombinants and variants might be expected to include immune surveillance and the ability to infect and persist through the complex life cycle of EBV. Immune surveillance would be expected to correlate with MHC type since functional epitopes will vary according to the presentation on MHC. Since predominant MHC types differ between racial groups and geographically, this could be a major factor in world wide variation of EBV. Many epitopes for CTL surveillance have been mapped in EBV antigens and correlated with MHC type. There is some clear evidence for epitope selection based on immune surveillance [28,29,30,31,32,33,34,35]. So far, little is known about the extent to which EBV selection *in vivo* may be affected by other polymorphisms that could affect, for example, ability to promote cell proliferation or viral replication. 

There are several examples of deletions of part of the EBV genome in endemic BL cell lines. The best characterized of these are the approximately similar deletions in P3HR1, Daudi, Sav, Oku and Ava BL cells [36] that remove EBNA2, most of BHLF1 and the C terminal part of EBNA-LP. The significance of these was interpreted as a mechanism for avoiding EBNA2 antagonism of c-MYC function [36] in the tumor cells. Further investigation indicated that enhanced expression of BHRF1 (the anti-apoptotic, viral BCL2 homologue) as a result of the deletion could also play a role in some BL cells harboring EBV with this type of deletion [37]. 

Some other well characterized EBV deletions are in the Raji BL cell line. Two separate deletions result in a loss of EBNA3C and some genes essential for lytic DNA replication [38]. Complementation of the defective lytic cycle genes by expression of BALF2 was sufficient to restore lytic DNA replication in Raji cells [39]. As mentioned above, the B95-8 strain of EBV also has a deletion relative to most EBV isolates [9,10]; the B95-8 deletion removes many of the BART miRNA sequences and one of the lytic origins of replication but this does not seem to adversely affect lytic replication or immortalization of B cells. 

Rearranged, defective EBV genomes (known as het DNA) have been characterized in detail in the P3HR1 BL cell line [40]. The rearrangements cause constitutive expression of BZLF1 [41] and the resulting lytic cycle activation allows persistence of the defective genomes in the cell population. There have been reports of similar rearranged EBV genomes *in vivo* [42,43] but a recent study concluded that they are only present quite rarely *in vivo* [44]. 

## 4. Variation in EBV Genes

### 4.1. LMP1

The locus of variation that has attracted most investigation in relation to disease is LMP1, which seems to contain a higher degree of polymorphism than most EBV genes. LMP1 is a membrane protein which makes many interactions through its C terminal region that mediate important signal transduction. These interactions regulate NF-κB and cell survival in several ways, so it is easy to envisage how sequence variation could alter those processes. A key step forward in understanding the many points of polymorphism in LMP1 came with classification of LMP1 variants into 7 main groups [45] and development of a rapid heteroduplex assay that allows classification of LMP1 type in a large number of samples. The sequence variants of LMP1 relative to B95-8 were named Alaskan, China 1, China 2, Med+, Med-, and NC. This more general insight into LMP1 variation taking into account the whole LMP1 sequence [45,46,47] has also been used by many other groups [48,49,50,51,52,53,54,55,56,57,58,59,60] but there was generally little evidence for a specific disease association of variants. Similarly, investigation of variant LMP1 sequences in HIV patients in Switzerland identified polymorphisms (I124V/I152L and F144I/D150A/L151I), which were markers of increased NF-κB activation *in vitro* but were not associated with EBV-associated Hodgkin Lymphoma [61].

It is still not clear how many EBV variants are present within one individual or even whether being infected offers immune protection against acquiring additional EBV strains. Studies using the heteroduplex assay confirmed that individuals can be infected with multiple variants [62,63]. Multiple LMP1 variants can be found in people with infectious mononucleosis [64], Hodgkin Lymphoma or NPC [65] and there is also evidence from people who are immunosuppressed, for example AIDS patients, for infection with multiple EBV strains [66,67]. Based on LMP1 analysis, variants differ in abundance between throat wash samples and peripheral blood samples in a variety of conditions [63,68,69,70]. Evidence for a specific variant of LMP1 being involved in a cancer could be provided by finding selective presence of that allele in cancer cells relative to the virus in the saliva or peripheral circulation. This is exactly what was discovered in an analysis of an NPC patient [62] but the interpretation made was in the context of evasion of immune surveillance of the LMP1 in the MHC haplotype of the patient rather than specifically enhanced transforming activity of the LMP1. 

Interest in LMP1 variation and function was stimulated by reports that a variant with a 30 bp deletion (Cao LMP1) isolated from an NPC tumor had a greater transforming activity than the reference LMP1 [71,72]. There are many points of sequence difference between Cao LMP1 and the reference B95-8 protein but attention was focused on the 30 bp deletion in Cao LMP1. The 30 bp deletion (amino acids 346–355) includes part of C terminal activating region 2 (CTAR2, amino acids 351–386) of LMP1. CTAR2 [73] mediates signaling to NF-κB and AP-1 but the 30bp deletion does not alter the parts of LMP1 that activate NF-κB [74]. An analysis of 249 patients in Taiwan showed that patients with Cao CTAR2 variant had an increased risk of distant metastasis compared with non-Cao variant [75]. The Cao CTAR2 was also a negative predictor for overall survival and post-metastasis disease-specific survival in that series. Another study indicated enhanced ability in tumor derived variants to activate Erk kinase and induce c-Fos [76], although that was accounted for by G212S or S366T rather than the 30bp deletion. 

Since a previous review [2], many groups have investigated the presence of the 30bp deletion LMP1 in normal carriers [77] or a variety of EBV associated diseases [52,78,79,80,81,82,83,84,85,86,87]. A rarer 69 bp deletion in the C terminus has also been studied [88]; it was reported to have a reduced ability to activate the cell AP1 transcription factor [89]. In general it is clear that these variants are widely distributed with somewhat different frequencies in different parts of the world but in most studies there was no evidence for a specific association with disease. Other reports of LMP1 variation associated with specific populations and disease contexts, for example gastric and oral carcinoma [90,91,92,93] have mostly lacked sufficient numbers or control samples to interpret in the context of the relationship between variation and disease but some studies have suggested an association with disease [94]. 

### 4.2. LMP2A

Low levels of LMP2A enhance cell survival but high levels of LMP-2A can also interfere with signaling from the B cell receptor by binding of lyn and fyn tyrosine kinases to the hydrophilic *N*-terminal part of LMP2A. Sequence polymorphism has been described in the N terminal region but it did not affect the key phosphorylated residues [95]. Although sequence polymorphism has been detected in isolates from various parts of the world and various diseases [96,97,98,99], there is no evidence for a disease association at present. Sequence variation is present, for example in South East Asia and New Guineau [100], in mapped epitopes for class I restricted cytotoxic T cells but this is not sufficient to prevent the possibility of LMP2A being a target for immunotherapy of EBV associated cancers [101,102]. 

### 4.3. EBNA1

EBNA1 proteins frequently differ in size due to variation in the length of the gly-ala repeat but further differences in the unique parts of EBNA1 have been used to define P (prototype, B95-8) and V (variant) EBNA1, which differ at about 15 amino acids [103]. These each have two subtypes defined by the amino acid at position 487 (P-ala, P-thr, V-pro and V-leu). In the initial report [103], P-thr was most frequent in peripheral blood lymphocytes of African and American samples and in African tumors but most American EBV associated lymphomas had V-leu EBNA1. A subsequent report confirmed the subtypes but found no association with lymphoma [104]. The variation might affect immune recognition of EBNA1 [105,106,107] and has been noted in Chinese NPC samples [108,109,110,111] but there is no substantial evidence of disease association. It has been reported that the V-val subtype of EBNA1 has increased ability to activate the enhancer functions of oriP in transfection assays [112,113].

### 4.4. EBNA2

Many groups have confirmed the classification of EBV into Type 1 and Type 2 strains (also known as A and B types) but there is little evidence for a disease relationship to the types. One study found that Type 1 EBV was more likely to cause infectious mononucleosis than Type 2 EBV [114] but a second investigation found no significant difference [63]. Although Type 2 EBV is prevalent in the same sub-Saharan region of Africa as endemic BL, the frequency of Type 1 or Type 2 EBV in BL from that region seems to reflect the incidence in the population rather than a specific type association [1]. In Argentina, EBV-1 was present in 76% of healthy carriers, EBV-2 in 15%, and co-infections with both types in 7% [77] but in Australia 98% of infections were Type 1 [115]. Some subtype variation of EBNA2 has been noted in Chinese EBV associated carcinomas [116] and in European lymphomas [26] but there is little evidence that it is related to disease.

### 4.5. EBNA3 Family

Polymorphism in the EBNA3 genes is considered to be linked to EBNA2 type variation [20] but additional sub-variation in EBNA3A and 3C has been noted with no relationship to disease [117,118]. 

Since EBNA-3B is not required for immortalization by EBV, variation in EBNA-3B was originally considered mainly in the context of immune surveillance. For example [119], polymorphisms of EBNA-3B (called EBNA4 in that publication) were found to be frequent in EBV-associated Hodgkin’s Lymphoma, Gastric carcinoma, and AIDS-lymphoma but not related to patients' HLA-A11 status. Sequence variation in EBNA-3B is now being re-examined since it has been realised that EBNA-3B acts as a tumor suppressor gene [3]. Loss of function mutants of EBNA-3B have much higher B cell transforming activity than wild type and a propensity to cause DLBCL in a mouse model [3]. It will therefore be important to determine which of the many polymorphisms in EBNA-3B affect its function so as to increase the viral transforming activity. 

### 4.6. Other Latent Cycle Elements

The EBER RNAs are strongly expressed in all types of EBV associated cancer. The extent of sequence variation in EBERs is quite small and has been linked to the Type 1/Type 2 EBNA2 status [26,120]. A recent report identified some new variants in Chinese samples [121]. At present there is little information on sequence variation in the BART or BHRF1 miRNAs, although attention has been drawn to a G155849A polymorphism near the RPMS1 open reading frame in the BART region of EBV from EBV associated with NPC in China [122]. Variation in promoter activity affecting expression in reporter assays has also been reported for the Cp and Qp promoters in EBV isolates from Chinese NPC samples [123] but there is no evidence for a role in disease. In contrast, it is clear that the number of copies of the major internal repeat (IR1), which contains the Wp promoter, does affect the ability of EBV to express EBNA2 and EBNA-LP efficiently and has a major effect on transformation efficiency [124].

## 5. Lytic Cycle

BZLF1 is the transcription factor that initiates the lytic cycle reactivation in B cells and its promoter is tightly regulated since it mediates the switch from latency to the lytic cycle. There has been considerable interest in BZLF1 variants that might affect its function or expression [125,126,127]. Although there are suggestions of variant sequence in Zp correlating with disease [128] further functional analysis would be required to substantiate this. 

Natural variation in the BZLF1 protein sequence has been described in Chinese isolates but showed no relationship to disease [129,130]. Variation in the BZLF1 dimerization sequence was studied using synthetic peptides but found to have only a small effect on its activity [131]. Polymorphism has also been noted in BRLF1, the EBV transcription factor which cooperates with BZLF1 to activate the early lytic cycle genes. Although a possible disease association was noted, this would require substantiation with more samples and controls [132]. 

Natural variation has also been reported in the early lytic cycle genes BHRF1 [133] and BNLF2a [134]. BHRF1 is similar to BCL2 with anti-apoptosis activity and BNLF2a has a role in immune evasion, reducing cell surface HLA class I levels. Protein function was not affected by these sequence polymorphisms. In an earlier study, sequence variants of the BHRF1 protein were identified [135] but no effect of the variation was found in the ability to protect against apoptosis induced by cis-platin. 

BARF1 is expressed in the early lytic cycle in B cell reactivation but is also expressed in NPC cells. It is secreted and binds to Colony Stimulating Factor 1 (CSF-1), inhibiting the binding of CSF-1 to its receptor [136]. Sequence polymorphism has been identified in BARF1 in Indonesian and Chinese samples [137,138] but most likely reflects natural selection of EBV strains unconnected to carcinogenesis.

The late lytic cycle gene gp350 encodes a surface glycoprotein on the EBV particle which is the target of neutralizing antibodies for EBV infection. Sequence variation of this protein has been identified but again appears to show geographic restriction rather than tumor specific polymorphism [139,140].

## 6. Functional Difference between Type 1 and Type 2 EBNA2

EBV Type 1/ Type 2 classification is defined by the EBNA2 sequence as this is the most divergent locus in the EBV genome with just 54% identity in the primary amino acid sequence. The most important biological and functional difference between the two viral types is that Type 1 EBV immortalizes B cells *in vitro* much more efficiently than Type 2 EBV [21].When LCLs are transformed with Type 1 EBV, they grow more quickly and to a higher saturation cell density in comparison to Type 2 transformants [21]. This difference in *in vitro* transforming efficiency between Type 1 and Type 2 EBV has been mapped to the EBNA2 locus [141]. When a Type 2 P3HR1 EBV strain was engineered to carry a Type 1 EBNA2 sequence, this virus gained the Type 1 immortalization phenotype [141]. In contrast, EBNA3 type does not affect the immortalization ability of the virus, as replacing the Type 2 EBNA3 gene locus with corresponding Type 1 sequences in the P3HR1 EBV genome shows no difference in primary B lymphocyte growth transformation [142,143].

This *in vitro* transformation phenotype of Type 1 and Type 2 EBV also correlates with tumor formation frequency in SCID mice that were inoculated intraperitoneally with Type 1 or Type 2 *in vitro*-transformed LCLs [144,145] . In fact, similar rates of tumor induction were observed for EBV-LCLs generated *in vitro* with a wild-type Type 1 strain or with a Type 2 P3HR1 strain carrying a Type 1 EBNA2 in the SCID mice model [145].

More recently, a transfection assay with an LCL (EREB2.5) infected with EBV containing conditional EBNA2 function was used to compare the abilities of Type 1 and Type 2 EBNA2 to maintain cell proliferation [146]. Type 1 EBNA2 maintained the normal growth of the cells but the Type 2 EBNA2 did not, providing a simple cell growth assay for this aspect of EBNA2 activity. The reduced proliferation in cells expressing Type 2 EBNA2 correlated with loss of expression of some cell genes that are known to be targets of Type 1 EBNA2. Microarray analysis of EBNA2 target genes identified a small number of genes that are more strongly induced by Type 1 than by Type 2 EBNA2, and one of these genes (CXCR7) was shown to be required for proliferation of LCLs. The EBV LMP1 gene was also more strongly induced by Type 1 EBNA2 than by Type 2, but this effect was transient. The results indicated that differential gene regulation by EBV Type 1 and Type 2 EBNA2 might be the basis for the much weaker B-cell transformation activity of Type 2 EBV strains compared to Type 1 strains [146].

To map the part of the EBNA2 protein responsible for the enhanced cell growth activity of Type 1 EBNA2, the effect on EREB2.5 cell growth of the chimaeras of Type 1 and Type 2 EBNA2 was tested in the EREB2.5 cell growth assay [147]. Although the major sequence differences between Type 1 and Type 2 EBNA-2 lie in *N*-terminal parts of the protein, the superior ability of Type 1 EBNA-2 to induce proliferation of EBV-infected lymphoblasts was found to be mostly determined by the *C*-terminus of EBNA-2. Substitution of the *C*-terminus of Type 1 EBNA-2 into the Type 2 protein was sufficient to confer a Type 1 growth phenotype and Type 1 expression levels of LMP-1 and CXCR7 in the EREB2.5 cell growth assay. Within this region, the RG, CR7 and TAD domains were the minimum Type 1 sequences required. The results indicated that the *C*-terminus of EBNA-2 accounts for the greater ability of Type 1 EBV to promote B cell proliferation, through mechanisms that include higher induction of genes (LMP-1 and CXCR7) required for proliferation and survival of EBV-LCLs [147].

The physiological significance of the Type 1/Type 2 variation is not known at present. One interesting speculation is that Type 2 EBV might be favoured in conditions of chronic immune activation. This might be present in the parts of Africa where Type 2 EBV is most abundant due to other co-infections including malaria. Other situations where detection of Type 2 seems relatively common also involve disturbance of the immune system, for example in AIDS patients. 

## 7. Conclusion—Continuum of Variation or Specific Types?

At present EBV strains worldwide can be classified into Type 1 and Type 2 but these do not appear to be genetically linked to the other patterns of virus sequence variation described. There is clearly a substantial level of inter-typic recombination and there may yet be other points of natural variation in the virus that have not been analyzed. New techniques for rapid sequencing of the virus genome that will allow determination of hundreds of EBV sequences are being developed and it is likely that these will give a much clearer understanding of what wild type EBV is, and whether there is an association of specific virus variants to human disease. Renewed interest in an EBV vaccine will also be aided by the understanding of natural variation that will be revealed by current high throughput sequencing for EBV. 
